# Errors in Commentary

**DOI:** 10.1001/jamanetworkopen.2025.34156

**Published:** 2025-08-28

In the Invited Commentary titled “Challenges of Economic Evaluations of Unreimbursed Care Models,”^1^ published June 23, 2025, there was an error in the fifth paragraph of the commentary. The original article stated that costs in a study by Spellberg et al^2^ were “calculated using all patient encounters derived from the Los Angeles County Department of Health Service, which are costs across all Los Angeles county hospitals, not only LA General.” This should have read “using all inpatient encounters at LA General (ie, including surgical patients and other encounters not covered by the SAH [Safer@Home] program).” This article has been corrected.^1^
